# Validation and comparison of virtual reality and 3D mobile games for cognitive assessment against ACE-III in 82 young participants

**DOI:** 10.1038/s41598-024-75065-1

**Published:** 2024-10-13

**Authors:** Yesoda Bhargava, Ashwani Kottapalli, Veeky Baths

**Affiliations:** Cognitive Neuroscience Lab, Department of Biological Sciences, BITS Pilani K. K. Birla Goa Campus, Goa, 403726 India

**Keywords:** Virtual reality, Cognitive assessment, Gamification, Z-score analysis, Cognitive impairment, Dementia, Ecological validity, Validation, Mobile Games, Regression Analysis, Correlation, Early detection, Cognitive ageing, Dementia

## Abstract

Current medical and clinical ecosystem for dementia detection is inadequate for its early detection. Traditional cognitive assessments are introduced after cognitive impairment has begun to disrupt the real-world functioning of the person. Moreover, these tools are paper-pen based and fail to replicate the real-world situations wherein the person ultimately lives, acts and grows. The lack of tools for early detection of dementia, combined with absence of reliable pharmacological cure compound the problems associated with dementia diagnosis and care. Advancement of technology has facilitated early prediction of disease like cancer, diabetes, heart disease, but hardly any such translation has been observed for dementia or cognitive impairment. Given this background, we examine the potential of Virtual Reality (VR) and 3D Mobile-based goal-oriented games for cognitive assessment. We evaluate three games (2 in VR, one in mobile) among 82 young participants (aged 18–28 years) and compare and contrast the game-based results with their Addenbrooke Cognitive Examination (ACE-III) scores. Three main analysis methods are used: Correlative, Z-score and Regression analysis. Positive correlation was observed for ACE-III and game-based scores. Z-scores analysis revealed no difference between the two scores, and stronger statistical significance was found between game scores and cognitive health factors like age, smoking compared to ACE-III. Specific game performances also revealed about real-world traits of participants, like hand-use confusion and direction confusion. Results establish the plausibility of using goal-oriented games for more granular, time-based, and functional cognitive assessment.

## Introduction

Dementia is a global public health issue^1^. Worldwide there are 57.6 million cases of Dementia according to the Lancet Neurology^2^ and this number is projected to reach 153 million by 2050 according to the Global Burden of Disease study published in Lancet Public Health^3^. In India, the cases are expected to rise to 13.33 million by 2050^4^. In the same timeline, 13 million dementia cases are expected in the US^5^ and 1.6 million in the UK^6^. The largest rise is expected in China at 48.99 million^7^. These estimates confirm that dementia will be a global public health challenge in the future.

This global challenge is compounded by the lack of a medical cure^8^, and the absence of clinical assessments that can indicate pre-dementia^9^ or estimate later-life dementia risk^10^. Given these clinical realities of dementia, it is prudent to monitor and assess cognitive abilities starting early adulthood so that abnormal cognitive deficits can be detected timely. Prior research confirms that cognitive decline begins in healthy educated adults in their second and third decade of life^11–16^.

This decline is corroborated by cross-sectional and longitudinal trends in cognitive decline^11^. Cross-sectional comparisons also reveal that continuous age-related decline in regional brain volume^17–21^, cortical thickness^22,23^, myelin integrity^24,25^ begin in the 20s.

In the context of clinical limitations surrounding dementia care, and the evidence of cognitive decline beginning in early adulthood, it is logical to initiate efforts for detecting cognitive decline starting from this age-group. Unfortunately, conventional neuropsychological assessments are unsuited for this purpose. Traditional neuropsychological assessments like Addenbrooke’s Cognitive Examination(ACE-III), Montreal Cognitive Assessment (MoCA), Mini-Mental State Examination (MMSE) are administered only after family members start noticing abnormal cognitive behaviour in the person, such as unusual forgetfulness, wandering, functional incapacities not explained by bodily limitations or ageing effects^26,27^.

Traditional neuropsychological assessments have additional gaps that make them incompatible for detecting cognitive decline early. Some neuropsychologists may not apply the rating instructions strictly, leading to inter-rater variability^28^. In addition, the existing neuropsychological assessments under-represent tests related to high-level cognitive skills^9^, and these assessments are conducted in highly structured, simplified and quiet lab environments^29^ due to which it is challenging to extrapolate their results to real-world functional cognition. Moreover, the lack of time component in these assessments limits insights into the processing speed of the players which is vital to detect pathological cognitive decline^30,31^.

Most importantly, traditional neuropsychological assessments do not advise about the person’s real-world behaviour^26,32–36^. As a result, their scores lack ecological validity^29,37–41^. In fact, neuropsychological tests’ performance explain only 5–21% variance in patients’ daily functioning^42^, therefore they risk poor dementia prognosis^29,43–45^. For early detection of cognitive decline, assessments that can inform about real world cognitive competence are important, because the ability to conduct life independently is the true indicator of cognitive and functional health.

Given the shortcomings of the traditional cognitive assessments for monitoring and tracking cognitive decline early in life-course, there is a need for alternative tools that can inform objectively about cognitive and functional abilities of the people. In this context, goal-based games appear relevant. Games like Space Fortress^46^, Train^47^, Professor Layton^48^, DigiSwitch^49^, Sushi Go Round, Portal 2^50^, League of Legends^50^, Taboo^51^, Minecraft^52^, Big Brain^53^ can measure a spectrum of cognitive abilities, such as general intelligence, working memory, perceptual speed, fluid intelligence, problem-solving ability, spatial skills, persistence and abstract reasoning. A different category of games, called the *serious games,* exists specially to detect cognitive impairments^54^.

In addition to the above-mentioned games in the mobile/2D, Virtual Reality (VR) games are also used for cognitive assessment. The immersive nature of VR facilitates 3D simulation of the real-world which can facilitate more active and realistic cognitive assessment as compared to the mobile-based games^37,55,56^. For e.g.,VR-based games can be used to assess competency in the activities of daily living (ADL)^57^ which is the most informative indicator of cognitive health and independent living^58–60^. Moreover, unlike the traditional cognitive assessments VR-based goal-oriented games tap into the reactions, reflexes, and response of the player and allow scoring them in a time-factored manner. Due to this, VR games can quantify not only the accuracy of the player reactions, reflexes and responses but also their spontaneity and speed, which is critical for assessing cognitive decline^31^. Furthermore, VR-based game environments provide powerful sensory stimulation^61^ that provides distinct advantage over simple puzzle games for cognitive assessment^62^. These properties of VR-based game environments make them particularly relevant for ecologically valid cognitive assessment.

The efficacy of VR-based cognitive assessment is also established by neurological evidence. For example, authors investigating the use of spatial navigational strategies using a VR navigation game found increased hippocampal gray matter to be associated with higher VR game score, but not with the RAVLT (Ray Auditory Verbal Learning Test ) or RO (Rey-Osterrieth Complex Figure Task)^63^. Similar finding was reported in another VR-based way-finding game wherein navigation ability as measured by the game performance was associated with hippocampal volume^64^. These studies point out that good and bad performances situated on VR-based goal-oriented games correlate with the brain-level differences. Therefore, VR-based goal-oriented games can serve as reliable indicators of cognitive ability. In fact, several studies have demonstrated that VR-games measuring cognitive abilities are sensitive to regional brain activation and functional connectivity and can discriminate between the good and bad performances, further corroborating their use for cognitive assessment^65–69^.

The promise of VR-based cognitive assessments is also supported by the research experiments in countries like Italy^26^, Singapore^70^, and Denmark^37^. However, no such study has been conducted in India. Given the rapid rise of dementia in India^71^, and lack of trained neuropsychologists^72^, research on evaluating the potential of goal-based mobile/VR games is desirable and timely.

In our study, we present and discuss the results obtained from a pilot study on VR and mobile games among 82 young participants (aged 18–28 years). The choice of young participants was motivated by evidence of cognitive decline beginning in the 20s^12–16^. Moreover, we also believe that testing in this group is a good starting point for evaluating the potential for game-based cognitive assessment before piloting it in the elderly group.

Using the pilot data, we compare the quantitative and qualitative insights obtained from game-based and ACE-III scores. Z-score and correlational analyses are used to compare the two scores, and multiple regression analysis is used to explore the association between the scores and the cognitive health factors. We also discuss cases wherein game-based scores informed about real-world behaviours of participants. Overall, our study provides a holistic analysis of game-based cognitive scores vis-a-vis with ACE-III scores, and also contributes to the methodology for similar research works.

In the next section, we describe in detail the background and context relevant to our work. Later, we present methodology for the pilot including study design, sample section, and statistical analysis. We finally conclude after presenting and discussing the results.

## Literature review

Scores from traditional neuropsychological assessments, such as Mini-Mental State Examination (MMSE), Montreal Cognitive Assessment (MoCA), Addenbrooke’s Cognitive Examination (ACE-III) cannot predict real-world cognitive and functional behaviours as the tasks or questions in these correspond very little with the activities of daily living^56,73,74^. These tools measure cognition in a singular and isolated manner whereas real-world tasks demand simultaneous and integrated response to multiple stimuli like visual, semantic, emotional, and prosodic^75,76^. Therefore, traditional cognitive assessment scores lack ecological validity^29,39,40,77^.

Empirically, this lack has been demonstrated in real-world experiments with neurological patients^39^. For example, in one real-world case, a 27 year old female sustained closed head injury during road accident. While the CT and MRI scans revealed frontal and temporal lobe contusions, her neuropsychological tests results reported no evidence of impairment in intellectual functioning, memory, language, perceptual judgement, executive functions, motor abilities, problem solving and cognitive processing skills. However, when she was tested in a shopping task in the real-world, she exhibited aimless behaviour and inability to locate the items on the list; she demanded immediate attention and uttered expletives in public and had no social inhibition. Moreover, she spent all the money for the task but did not purchase the items on the list. Such discrepancy between the neuropsychological test results and the real-world behaviour corroborates that the former lack ecological validity.

To address the ecological validity gap, two methods currently exist. One method uses a *vector-based* approach. In this method, a vector containing the neuropsychological assessment scores and inputs from patients’ caretakers and family members is used to indicate the overall neuropsychological health of the patient^39,77,78^. Theoretically vector-based approach provides greater information about the real-world cognitive and functional abilities of the patients as compared to the cognitive assessments alone. Hence, it is more ecologically valid. Practically though, vector-based approach has operational limitations; its scalability is hindered by the time required for collecting and analysing data from other caretakers and family members. The method is useful for monitoring the progress of patients with cognitive deficits but is unsuited for early detection of cognitive deficits.

In the second method, researchers compute the proportion of variance explained by the neuropsychological assessment scores in the activities of daily living (ADL), such as telephone dialling or currency counting^38^. Through this method, a direct evaluation of the ecological validity of the scores is obtained. But it is impossible to scale this approach to encompass all the ADLs. Thus, vector-based and variance-based approaches address the ecological validity gap, but it is impossible to scale them on large scale. Moreover, the advantages they confer over traditional assessments are applicable only to existing patients. Therefore, both vector and variance-based approaches are unsuited for early detection of cognitive decline.

Given these limitations, alternative tools for ecologically-valid assessment of cognitive abilities are desired. Goal-oriented games appear favourable in this context. Geriatric health care centres, dementia care NGOs and memory clinics use such games to exercise patients’ memory, improve their language expression and develop reasoning abilities. The long-term impact of such engagement on cognitive and functional abilities is hardly monitored, nor is it used to inform the rehabilitation strategies. Manual supervision and scoring is a plausible reason for this drawback. Furthermore, scores based on these board or offline games are based on correct and incorrect responses which seldom provides insights into the cognitive strengths and weaknesses of the participants. These challenges in the conventional brain engagement games can be resolved by software-based end-to-end goal-oriented games.

Software-based games can inform the real world navigation skills and recall as observed in a study of Alzheimer’s Disease patients^79^. Games like Big Brian Academy, Kitchen and Cooking, Minwii are used to treat cognitive impairment^80,81^ and Wiifit and Wii Sports to deal with movement disorders^82^. If these games can improve the real-world cognitive and functional abilities of the people, then game-performance can be used to estimate these abilities. Thus, such games can provide ecologically-valid cognitive assessment and also facilitate automated scoring useful for long-term tracking and monitoring of cognitive abilities^83^.

Software-based end-to-end goal-oriented games in the VR platform enhance cognitive testing by introducing realism and immersion in the game environments. Unlike the two-dimensional games in the mobile or web platform, VR-based games can simulate real world and mimic the cognitive engagement akin to the real world tasks^56^. For instance, participants’ ability to perform a diverse range of instrumental ADLs (IADLs) such as transportation, telephone use, meal preparation, medical management, financial management, housekeeping, laundry, shopping can be tested easily in the VR environment^55,84^. Even without duplication of the real-world environments, animated game environments in VR can test multiple cognitive skills due to VR’s immersive, interactive and engaging nature.

VR-based games can directly assess total encoding, recall, and delayed recall^85^. In fact, scores from VR-based games like Virtual Reality Day-Out Task (VR-DOT), a fire evacuation game, showed strong correlation ($\rho = 0.32$, $p-value =0.001$) with the standard MMSE and Bristol ADL scores. Similar strong correlations were observed between VR-based games and real-world navigation abilities^86,87^. These correlational results on VR-game scores and real-world abilities testify that VR-based games can provide ecologically valid cognitive assessment.

Indirect evidence of VR-based games for cognitive assessment is found in a cluster randomized controlled trial^88^, in which 102 older adults were divided into cyber-cycling (VR + cycling) group and traditional exercise group. Superior cognitive effects were observed in the cyber-cycling group. If VR-based game training can provide cognitive benefits, then cognitive abilities can also be estimated directly from VR game performance. In fact, cognitive benefits from other games^89–94^ and improved MMSE score post VR-based training^95,96^ endorse that VR and mobile-based game-scores can be adopted to directly estimate the real-world relevant cognitive and functional abilities.

Even neurological evidence has shown that games which demand concentration, hand-eye coordination and distraction-avoidance lead to electrical activity and activation of brain regions^89^. VR-based goal-oriented games have been shown to be associated with brain areas such as pre-frontal cortex^97^, retrosplenial cortex^69^, dorsolateral pre-frontal cortex^98^, hippocampus^63,64,68,99^, basal forebrain^68^, ventrolateral pre-frontal cortex^67^ in both healthy and cognitively impaired people. Evidence for activation of the frontal, parietal, temporal and occipital lobe also prove that VR-based goal-oriented games engage regions associated with cognitive function^61,100–102^. Even brain waves are associated with the VR-based games^103^. Clearly, VR-based goal-oriented games tap into the brain regions and are also associated hippocampus, which is the main brain region to show early signs of deterioration before dementia onset^104^.

From the aforementioned evidence, it is clear that goal-oriented games do not superficially engage the player, instead the scores obtained from them can potentially estimate cognitive abilities. Moreover, the fact that VR-based goal-oriented games are more ecologically valid than the traditional assessment methods further lends credence to their use for functional cognitive assessment^105–110^. Due to these advantages, these software-based and VR end-to-end goal-oriented games must be examined for their capability to measure cognitive abilities across life-course. Motivated by the evidence linked to VR and mobile-based games for cognitive assessments, we conducted a pilot for VR games in India and describe our findings in this work. In the next section, we explain and justify the methodology used.

## Methodology

### Study design

The recruitment for the pilot was advertised through the university communication and all participants who responded within the duration of 3 weeks from the advertisement date were recruited. Inclusion criteria was: (a) familiarity with English or Hindi language, (b) cognitively healthy without any history of epilepsy, vertigo, or motion sickness risk, (c) intact motor and hearing abilities, and (d) willingness to complete questionnaires and interview part of the study. The exclusion criteria was: (a) disability, (b) neurological deficits of vision, hearing, speech or motor skills, (c) severe cognitive impairment or psychiatric disturbances, (d) terminal conditions or (d) under serious medication.

### Ethical considerations

Written informed consent to participate in this study was provided by all the participants. Procedures contributing to this work comply with the ethical standards of the relevant national and institutional committees on human experimentation and with the Helsinki Declaration of 1975, as revised in 2008. All procedures involving human participants/patients were approved by the Human Ethical Committee of BITS Pilani Goa Campus (Ref. No. HEC/BITS Goa/2023-2026).

### Study details

This is an experimental observational cross-sectional study. Each participant session was divided into three phases: Pre-game phase: Participant information acquisition, Game Administration phase, and Post-game phase: Participant feedback and interview. These are described next.

#### Pre-game

Each participant was briefed about the study and a written consent was obtained. A unique identifier (UID) was given to each participant to ensure data privacy and confidentiality. Personal information such as demographic details (age, gender, education) and lifestyle (physical activity, sleep, smoke, alcohol consumption, social interaction, artistic inclinations) was collected. Following this, mindfulness score were obtained using the Kentucky Inventory of Mindfulness Skills (KIMS)^111^. It is a self-report inventory used to assess mindfulness and its scores are based on four aspects: Observation, Ability to Describe, Act with Awareness, and Non-judgmental behaviour. Because increased mindfulness is related to greater cognitive performance^112^, we measured mindfulness for each participant to account for confounding in cognitive performance.

Next, each participant was administered ACE-III. We chose ACE-III because it provides validated measures of cognitive performance in five domains, attention, memory, language, visuospatial skills, and fluency^113^. Scores from these individual domains provide richer insights about the cognitive profile of the person^114^. Due to this, ACE-III has a greater diagnostic ability and it is also less prone to the ceiling and floor effects^115,116^. Importantly, ACE-III provides superior insights about the functional cognition than both MMSE and Montreal Cognitive Assessment (MoCA)^117^. Given our focus on ecologically valid cognitive assessment, we chose ACE-III over MMSE and MoCA as a comparator for the game-based cognitive scores.

Three games designed by us were piloted: Navigation Game, Hand-Eye Co-ordination Game and the Memory Game. The first two are VR-based games, and the last one is Laptop/Tablet-based. The VR games are animated to instil a sense of wonder and excitement conducive for participation. The tasks in the games mimic the cognitive demands of the real world activities like walking, turning, catching, navigating, memorizing, attention, response inhibition, hitting etc. Therefore, it can be reasonably assumed that scores from the three games indicate real-world decision-making and cognitive engagement. Details about the game tasks can be found in the Supplementary Section.

The games were administered in the following sequence to avoid excessive VR exposure: Hand-Eye Co-ordination Game, Memory Game, and Navigation Game. Participants were shown video tutorial for the VR games and were made to play a mini-version of each VR-game to familiarize with the controller usage. For both the VR games, final score was based on the scores of the three trials, because score from single trial is not a reliable indicator of cognitive performance in novel game environment, like VR. In contrast, for the memory game, only one trial was conducted given the relatively simpler game environment. Scores from all three games (Table  1) were necessary to complete the participant profile and prerequisite for the analyses.

**Table 1:** Table showing the cognitive scores developed by us for our VR (immersive) and 3D Mobile / Tablet games.

**Game platform**	**Game name**	**Cognitive scores**
Virtual reality (immersive)	Navigation game	Processing speed, Attention and planning, Navigation ability, Visuospatial ability, Total land score, Fly Score
Virtual reality (immersive)	Hand-eye coordination game	Processing speed, Attention scores, Motor abilities, Total hand-eye score
Mobile or tablet	Memory game	Semantic memory score, Visual short-term memory score, Total memory score

For the memory game, we assessed only the short-term interval memory recall because of the time constraint. The three trials of the 2 VR games, 4 levels in the memory game, 3 feedback forms, and the interview took 1 hour for each participant. All these assessments were very important for our experiment. To avoid fatigue in the participant we decided not to include the long interval delayed recall. However, this was measured in the ACE-III test we administered to the participants. Also, the long-interval delayed recall was indirectly assessed when participants were interviewed to probe their game performance. Still, it was not quantified in this study, and is a limitation of this work. Going forward, the long-interval delayed recall can be assessed by asking the participants to recall the VR game environments explicitly by asking them to make a sketch map^69^, by gamifying as done in a route recognition VR game^118^ or by inclusion of a delayed memory recall component^65,119^.

Because the games developed by us were goal-based and involved activity, we could not include language and fluency as the cognitive domain for game-based assessment. Another reason was that most of the studies on early detection of cognitive impairment have pointed out that the deficits in memory and spatial navigation precede dementia onset by 1–3 decades^104^. Moreover, Salthouse et. al, in their work based on cross-sectional and longitudinal studies proved that memory, spatial visualization, speed begin to decline in the second decade of human life supporting that assessment of these abilities is crucial for early detection of pathological cognitive impairment^11^. Due to these evidence, we focused on executive function (attention, planning, processing speed), motor abilities, and short-term memory as the primary cognitive skills to be measured using the developed games. However, linguistic features are relevant for detection of cognitive deterioration, especially for early MCI^120^ and amnestic MCI patients^121^. In future, we hope to also incorporate the language and fluency aspects in the VR and Mobile based games.

A detailed conceptual explanation and mathematical formulation for each game score used in our study (Table  1) is provided in the Supplementary Section.

#### Post-game

**Fig. 1:**
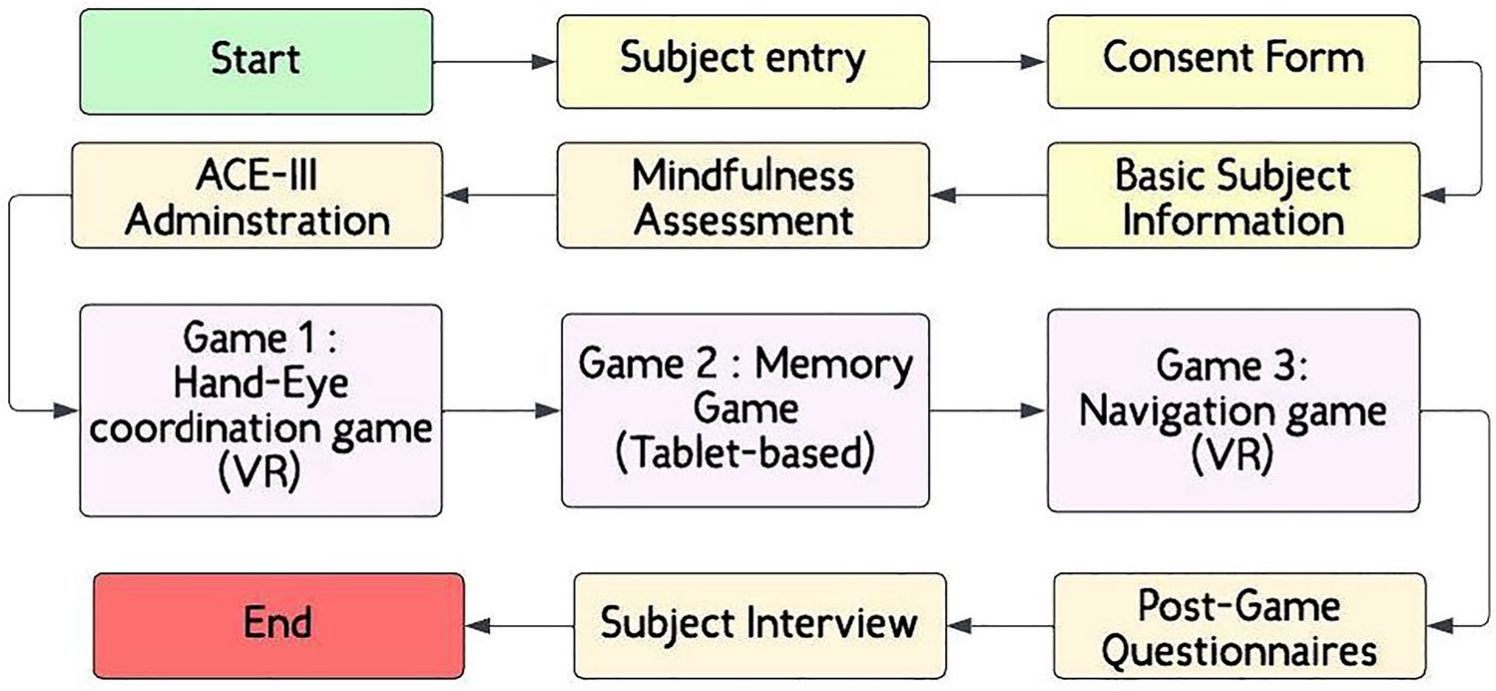
Flow chart describing the steps followed in the research process.

Post game administration, participants were allowed to rest for 1–2 minutes. Later, in order to obtain the feedback from the participants, we administered standard questionnaires to them: Virtual Reality Sickness Questionnaire^122^, Virtual Reality Presence Questionnaire^123^, and the System Usability Scale^124^. Post this, an interview was conducted to obtain contextual clarity about the participants’ feedback in the questionnaires. The entire process of pre-game, game administration and post-game took 1 hour (Fig. 1).

### Statistical analysis

#### Score comparison

Through our study, we wanted to assess the promise of the game-based cognitive assessment, therefore we compared the obtained game scores with the ACE-III scores. Thus, ACE-III total and its constituent scores (Attention, Memory, Visuospatial) acted as the comparator for the game-based scores. Following steps were undertaken to develop game-based counterparts to the ACE-III constituent scores:

1. Game-based Total Cognitive score (Eq. 1) acted as a counterpart to the ACE-III total.
  1 $$\begin{aligned} \begin{aligned} \text {Total Cognitive Score}&= \text {Land Score} + \text {Fly Score} + \text {Hand-Eye Score}\\&\quad + \text {Semantic Memory Score}\\&\quad +\text {Visual Short-Term Memory Score} \end{aligned} \end{aligned}$$
2. Game-based total memory score used as a counterpart to the ACE-III total memory score. (Details in the Supplementary Section titled Memory Games)
3. Fly score (Equation 6 in Supplementary Section) used as a counterpart to the ACE-III Visuospatial Score.
4. Sum of twice the Attention score (Equation 2 in Supplementary Section) from the Navigation game, and the average of Right (Equation 8 in Supplementary Section) and Left-Hand Attention (Equation 9 in Supplementary Section) from the Hand-Eye Coordination game used as a counterpart to the ACE-III Attention score. The Navigation game demanded greater attention and planning for obstacle avoidance as compared to the Hand-Eye Coordination Game; therefore attention score obtained from the former was doubled to give it more weightage in the total game-based Attention score (Eq. 2).
  2 $$\begin{aligned} \begin{aligned} \text {Total Game-Based Attention Score}&= 2AP_n + \frac{ALH_h + ARH_h}{2} \end{aligned} \end{aligned}$$

Thus, we compared the following scores in the ACE-III and games: Total Score, Memory, Attention, and Visuospatial. Given the different distributions for both the ACE-III and game-based scores, z-score comparison was used. We correlated the z-scores, used paired t-test and found out the 95% limits of agreement for the z-scores. The null and alternative hypothesis for paired t-testing were:

3 $$\begin{aligned} \begin{aligned} H_{0}&= \text {There is no difference in the z-scores for the ACE-III and game-based scores.}\\ H_{a}&= \text {There is a difference in the z-scores for the ACE-III and game-based scores.} \end{aligned} \end{aligned}$$

#### Linear regression

We also used multiple linear regression to understand the association between game-based scores and cognitive health factors. Previous works have regressed these scores on traditional neuropsychological assessment scores or vice-versa. This approach seems unfruitful as the game-based scores are validated against traditional neuropsychological scores which have several limitations.

Instead, we wanted to investigate the direct association of the game-based scores with the cognitive health factors (Eq. 4), as these factors are related to cognitive decline^125,126^. Insights on these associations provide direct evidence about the efficacy of game-based assessments for cognitive assessment and tracking.

4 $$\begin{aligned} \begin{aligned} \text {Game-Based Score}&= \beta _0 + \beta _1 * {\textit{age}} + \beta _2 * {\textit{gender}}\\&\quad + \beta _3 * {\textit{sleep}} + \beta _4 * {\textit{sleep quality}} \\&\quad + \beta _5 * {\textit{alc consumption}} \\&\quad + \beta _6 * {\textit{coffee}} + \beta _7 * {\textit{smoke}} \\&\quad + \beta _8 * {\textit{social interaction}}\\&\quad + \beta _9 * {\textit{observation ability}} \end{aligned} \end{aligned}$$

In total we regressed 8 scores: Final Navigation Score (FNS), Final Hand-Eye Score (FHS), Semantic Memory Retrieval Speed (SMRS), Visual Short-Term Memory Retrieval Speed (VSTMRS), Fly Score (FS), Navigation Game Attention (NGA), Hand-Eye Game Attention (HEGA), and ACE-III total. Gaming and technology-based factors were excluded from ACE-III regression because they were assumed to be unrelated to ACE-III in the context of the study. The regression coefficients for game-based models were interpreted and compared against those obtained in the ACE-III regression.

In addition to the statistical analyses, we also discuss specific situations in which game-based performance indicated real-world behaviour of the players, hinting at the scores’ ecological validity.

## Results and discussion

A total of 91 people participated in the pilot that was conducted in the months of May-June 2023. Out of 91 participants, 5 (UID = 1012, 1013, 1025, 1069 and 1084) were removed from the population-level analyses because of incomplete data. UID 1012 refused to play the third trial of the Navigation game due to dizziness and discomfort. UID 1025 played only 2 trials of the Navigation game and quit in the third trial due to discomfort. UID 1069 refused to play the Navigation game due to dizziness during the video tutorial. UID 1084 could not play second and third trial of the Navigation game due to cough and cold. UID 1013’s data for the last trial of the hand-eye co-ordination game was lost and could not be recovered. The game data for 4 participants (UID 1056, 1057, 1058, 1059) was lost during cloud server update and could not be recovered. Finally thus, data from 82 participants was used for analysis.

### Descriptive summary

Majority of the participants were young; the minimum age was 18 years, maximum was 29 years and the mean age was 20 years (Table  2). Out of the 82 participants 67 (81.71%) were males. Majority of the people were right handed (94%), 3 were left-handed, and 2 were ambidextrous. 79 participants were pursuing a graduate degree and 3 were pursuing doctorate. 44 (53.66%) reported drinking coffee, 17 (20.73%) people reported consuming alcohol (once a month alcohol). Three participants (3.66%) reported smoking and all of them also consumed alcohol once a month.

**Table 2:** Descriptive Summary of the sample and characteristics of the participants.

Variable	Scale/unit/statistic	Mean (95% CI)
Demographic data		
Age	Years	20.22 (19.76, 20.68)
Gender (Males)	Proportion	81.71 (67%)
Education Level	Degree	Graduate students
Sleep	Hours	6.66 (6.44, 6.89)
Sleep Quality	On the scale of 10	7.50 (7.12, 7.88)
Exercise	Frequency	Almost daily
Regular Coffee Consumption	Proportion	53.66% (44)
Regular Smoking	Proportion	3.66% (3)
Occasional Alcohol Consumption	Proportion	20.73% (17)
Gaming and Digital Experience		
Gaming Experience	On the scale of 5	4.06 (3.88, 4.24)
Digital Experience	On the scale of 5	4.60 ( 4.47, 4.73)
VR Experience	On the scale of 5	2.22 (1.97, 2.47)
Mindfulness scales based on KIMS		
Observation Abilities	Out of 60	39.21 (37.70, 40.72)
Acting with Awareness	Out of 50	29.26 (27.86, 30.65)
Traditional Neuropsychological Assessment		
ACE-III Score	Out of 100	93.40 (92.72, 94.08)
ACE-III Attention	Out of 18	17.56 (17.41, 17.71)
ACE-III Memory	Out of 26	23.26 (22.92, 23.59)
ACE-III Fluency	Out of 14	12.91 (12.65, 13.18)
ACE-III Language	Out of 26	24.58 (24.34, 24.82)
ACE-III Visuospatial	Out of 16	15.09 (14.87, 15.30)
Game-based Assessment		
Navigation game		
Land Score	Higher the better	13.07 (11.27, 14.87)
Fly Score	Higher the better	1.84 (1.66, 2.02)
Hand-Eye Game		
Hand-Eye Score	Higher the better	27.02 (24.38, 29.65)
Attention Left hand	Proportion	0.95 (0.94, 0.96)
Attention Right hand	Proportion	0.95 (0.93, 0.96)
Memory Game		
Semantic Memory Score	Higher the better	25.74 (22.84, 28.65)
Visual-Short Term Memory Score	Higher the better	13.55 (10.83, 16.27)
Total Memory Score	Higher the better	34.85 (39.30, 43.75)
Game-based Total Cognitive Score		
Total Score	Higher the better	81.14 (74.98, 87.30)
Game Feedback (VR and Tablet)		
VR-Sickness	Lower the better (Max score = 100)	14.48 (11.58, 17.39)
Realism	Higher the better (Max score = 49)	36.29 (34.75, 37.83)
Possibility to act	Higher the better (Max score = 28)	23.70 (22.99, 24.40)
Quality of interface	Higher the better (Max score = 21)	17.16 (16.54, 17.79)
Usability	Higher the better (Max Score = 100)	85.82 (83.51, 88.14)

68 participants (83%) reported doing planned physical activity, and the remaining 14 reported walking as their primary physical activity. Thus, almost all participants were physically active. The mean sleep duration of the sample participants was 6.66 hours, and the mean sleep quality was 7.5 out of 10. Although on average sleep duration and quality is adequate, 9 (11%) people reported difficulty sleeping.

On the scale of 5, participants reported high gaming (4.06) and digital experience (4.6) and low VR experience (2.22). In the case of mindfulness, out of maximum score of 50, the average score for Observation abilities was 39.21 and for Acting with Awareness was 29. Average score for ACE-III was high at 93.40/100. For its constituent scores like Attention (17.56/18), Memory (23.26/26), Fluency (122.91/14), Language (24.58/26) and Visuospatial Abilities (15.09/16) average scores were close to the maximum values. Given the younger population, higher scores are expected.

The average scores from game-based assessments are also shown in Table  2 but it is difficult to comment on all of them because the respective maximum scores are not known. Therefore, the average scores can be evaluated in the intra-participant context only. Whereas, average score for Attention Left and Right hand can be analysed categorically as it is a proportion. In the given sample, on an average the participants hit 95% of the incoming cubes accurately for both the right and the left hand, indicating relatively good attention for each hand.

As for the game feedback, participants rated VR-related sickness as low (14.48/100), and Realism in the game (36.29/49), Possibility To Act in the VR games (23.70/28), System Usability (85.82/100) and Quality of Game Interface (17.16/21) as high. The feedback for Realism (36.29/49) shows that participants equated realism to real-world objects in the game. However, we assert that realism in the context of VR games is a broad concept and is not limited to rigid duplication of the real-world. Instead it encompasses mimicking the cognitive load and engagement observed in the real-world. From this stand-point on realism, our games are reasonably real and the animated components engage and excite the user to play the game. Overall, the participants gave positive feedback on the VR games.

Before discussing the results of z-score and linear regression, we discuss specific observations from each of the games.

### Game score analysis

The highest game-based total cognitive score was 192 and the lowest was 17, mean was 81.15, and median was 81.65. The standard deviation of the game-based total cognitive score was 28. High total score values are due to the higher range of scores (Land Score Range = (2.2, 36.8), Hand-Eye Coordination Score Range = (1.59, 66.06), Fly Score Range = (0.084, 3.75), Semantic Memory Score Range = (6.2, 89.7) and Visual Short-Term Memory Score Range = (0.3, 56.5)).

The highest value for the navigation game was 20.82 (ID = 1023), the mean value was 13.07 (95% CI (11.27, 14.87)) and the minimum was 8.64 (ID = 1019). For the Fly component of the navigation game, the maximum score was 3.75 (ID = 1028), mean was 1.84 (95% CI (1.66, 2.02)) and the minimum was 0.084 (ID = 1005).

The highest score for the Hand-Eye Coordination game was found to be 66 (ID = 1007) and the lowest was 1.59 (ID = 1071); the mean score was 27.02 (95% CI (24.38, 29.65)). Most participants obtained >90% accuracy for both left and right hand hits, except for the 7 participants. Out of these 7, five were right-hand dominant (mean age = 20 years old), one was left-handed (23 year old), and the other was ambidextrous (27 year old). ID = 1071 reported the lowest hit accuracy (Right-Hand = 76% and Left-Hand = 73.5%) among the Right-Handed participants. His Observation score using the KIMS Mindfulness form was also lowest among the sample size (score = 28, sample average = 39.22). Thus, his accuracy scores for left and right hand may indicate his lower observation ability in general.

The ambidextrous person (ID = 1005), achieved the right hand accuracy of 74% and left-hand accuracy of 78.7%. The higher proportion of left-hand proportion confirms that the person was born left-handed, but the overall lower proportion of the right and left-hand accuracy validate his confusion in hand-use as confirmed by the participant in the interview. The participant explained that he is naturally left-handed but due to parental pressure, he had to learn to use his right-hand. Due to this coercion, he does not have fully developed decisiveness in hand-usage. This confusion is exemplified in the hand-eye coordination game wherein he consistently chose wrong hammer for hitting the cubes.

ID 1005 also reported poor performance in the Fly segment of the navigation (fly score (0.084)). There appears to be a pattern in this case because the the participant consistently performed below average in tasks associated with directional hand movements (fly segment and the hand-eye coordination game). Noteworthy however is that he obtained 95.5 in the ACE-III which is greater than the group average of 93.40. According to the ACE-III, ID 1005 performed better than average person in the sample, but in the game-based assessments similar conclusion cannot be drawn. ID 1005 was unique due to their issues with handedness; while the game-based assessments could capture this uniqueness the ACE-III assessments could not. This particular case highlights the extremity of the discrepancy between ACE-III and game based assessments, and the potential of the latter to tap into cognitive traits.

We also looked at the peculiarities in the total number of wrong hits in the hand-eye coordination game. There could be three types of wrong hits: cube hit by wrong colored hammer (Type 1), cube hit with correct hammer but in the wrong direction (Type 2), or both Type 1 and Type 2 simultaneously. For ID 1002, it was noticed that all wrong hits were Type 2. Later, in the interview when we pointed out this observation, the participant confirmed they are usually confused and indecisive about directions in the real-world. From this particular case, we realized that the nature of incorrectness in the game also revealed about the participant’s cognitive and behavioural attributes, which could not be obtained from their ACE-III results.

The relationship of game-based scores from ID 1005 and 1002 with their real-world behaviour demonstrate the potential of VR and 3D goal-oriented games for functional cognitive assessment and its ecological validity.

For the Semantic Memory Game (Fig. 4(a) and 4(b) in Supplementary Section) the highest score was obtained by ID = 1030 (Score = 89.7), mean score was 25.74 (95% CI (22.84, 28.65)).The lowest score was observed for 1070 ID (score = 6.2). IDs 1005 and 1071 also scored very low in the Semantic Memory Game (Score = 9.1 and 7.4 respectively). ID 1005 identified correct answers in 2 attempts but the time taken was 32 seconds (Office environment) and 16 seconds for the Kitchen environment. For ID 1071, the person took 21 seconds and 3 attempts to identify the 2 correct answers in the Office environment, and 24 seconds to correctly identify answers in the Kitchen environment in 2 attempts. Due to higher amount of time, ID 1005 and 1071 scored low on the semantic memory game, whereas, their ACE-III Memory scores were very high (ID 1005: 25/26 and ID 1071: 24/26). These differences in the ACE-III and game-based memory performances are due to the use of time factor in game-based scoring (Table 1 in Supplementary Section). The time-factored scores provide a higher level of discrimination across the participant performances and help in obtaining insights on retrieval and information processing speed which are useful for informing memory loss^127^.

In the Visual Short-Term Memory game, highest score was obtained by ID 1001 (score = 56.5). This participant was able to detect the changes in 2 attempts for both the Color change (2 seconds) and Object Disappearance Task (10 seconds). ID 1005 had the lowest score (0.3, group average = 14.22) and ID 1071 obtained a score of 0.6. ID 1005 and 1071 took 5 attempts but could identify only one correct answer for both the Color change and Object Disappearance Task.

All participants, in general, obtained higher score for Semantic Memory game as compared to the Visual Short-Term memory score (paired t-testing, mean difference = 6.09, 95% CI = [8.24, 16.14], p-value < 0.0001). Overall, the game-based memory scores provided information about the speed and precision of the semantic memory and visual short-term memory recalls. Such insights are impossible to obtain from the ACE-III and pen-paper based neuropsychological assessments.

In the ACE-III assessment, memory is assessed by asking about address, objects name recall, and names of famous personalities. While location address and objects recall are vital for real world functioning; engaging and searching for objects also forms an important cognitive skill for the real-world operation. Our games provided an avenue to engage with real world objects in a 3D game environment, thus making the assessments more realistic and engaging to play.

From the overall analysis of the game-scores, we noticed that some behaviour patterns could be captured by the game-based assessments and not the ACE-III assessments. We also realized that ID 1005 stood out unique as they scored low in the game compared to the overall sample, and given their extreme smoking habits (21 cigarettes per day), would form the best candidate for longitudinal assessment of cognitive and behavioural habits.

Next, we present and discuss the results of comparison of ACE-III and game-based z-scores.

### Z-score analysis

Table  3 shows the results of z-score comparison for the ACE-III and game-based scores. The correlation between game-based total cognitive score and ACE-III total, is 0.114. Although the correlation magnitude is low, a positive value indicates that ACE-III total and Total Game Scores are aligned with each other. The paired t-test statistic is not statistically significant (Mean Difference: 0, 95% CI (− 0.292, 0.292)). Therefore, the null hypothesis that the difference in the z-scores is zero is not rejected (Eq. 3).

**Fig. 2:**
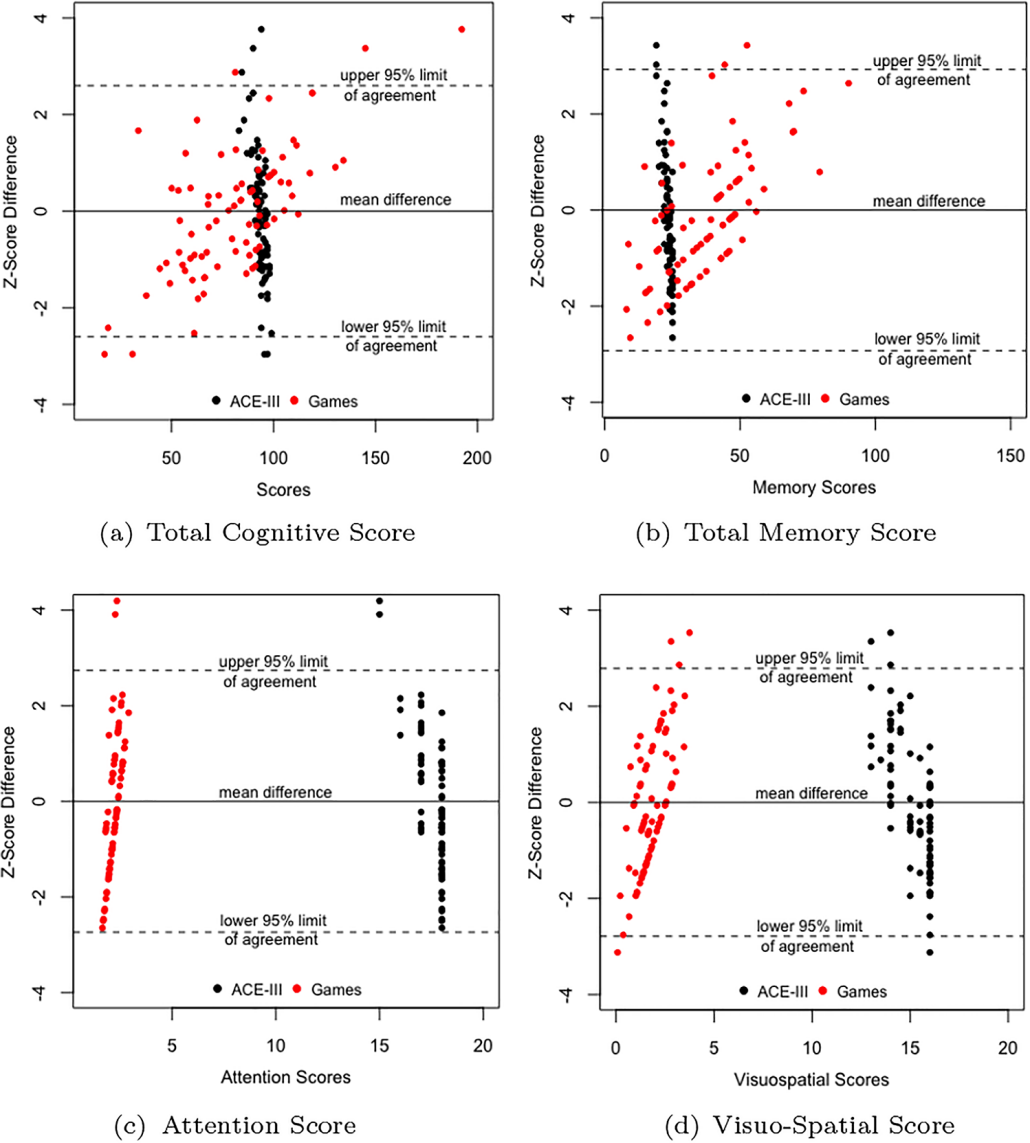
Figures showing the 95% limits of agreement for ACE-III and game-based z-scores.

The correlation and z-score analysis indicate that the ACE-III total and game-based total cognitive score obtained for each participant in the study are not different statistically, supporting game-based cognitive assessment used in the experiment. The 95% limits of agreement for the two z-scores are ($-$2.60, 2.60). Thus, for a given score, the z-scores are expected to be 2.60 standard deviations below to 2.60 standard deviation above for most participants. These limits are shown as horizontal lines in Fig. 2a. The figure also shows that ACE-III scores are relatively less spread out as compared to the game-based scores. Thus even in the same age-group, game-based scores differ unlike the relatively stable ACE-III scores. This indicates that game-based scores have superior discriminating power than the ACE-III scores.

**Table 3:** Summary of z-score comparison of ACE-III and game-based scores.

Score type	Correlation (p-value)	Paired Mean Difference (95% CI), p-value	95% Limits of Agreement
Total	0.114 (0.31)	0 (− 0.292, 0.292), $\approx$ 1	($-$2.60, 2.60)
Memory	− 0.114 (0.31)	0 (− 0.328, 0.328), $\approx$ 1	($-$2.92, 2.92)
Attention	0.025 (0.82)	0 (− 0.307, 0.307), $\approx$ 1	($-$2.73, 2.73)
Visuospatial	− 0.0117 (0.91)	0 (− 0.313, 0.313), $\approx$ 1	($-$2.78, 2.78)

The correlation between game-based memory and ACE-III memory z-scores is − 0.114. The reason for negative magnitude are those cases which perform well in the ACE-III memory tasks but compared to their ACE-III memory scores, the game-based memory scores are low. One reason for the low performance scores could be inclusion of time factor in computing the game-based memory scores. The paired t-test for the difference in z-scores of game-based memory score and ACE-III memory score is not statistically significant (Mean Difference = 0, 95% CI (− 0.328, 0.328)). Thus the null hypothesis of no difference is not rejected, implying that game-based memory scores are not radically different from ACE-III scores in the given data. The 95% limits of agreement for the memory z-scores is ($-$2.92, 2.92) (Fig. 2b). From Fig. 2b we observe that the memory scores from ACE-III lie very close to each other (95% CI for mean = (22.92, 23.59)) whereas memory scores from the games are relatively more dispersed (95% CI: (39.30, 43.75)).

For the attention z-scores, a correlation of 0.025 was observed; the paired t-test was not statistically significant (Mean Difference = 0, 95% CI (− 0.307, 0.307)) and the p-value was approximately 1. Thus, the null hypothesis of no difference in the z-scores cannot be rejected implying that the z-scores from the two different modalities are not statistically dissimilar in the given data. The 95% limits of agreement for the z-scores is ($-$2.73, 2.73) and are also shown in Fig. 2c. The figure shows that attention scores from ACE-III are discrete whereas from the games form a continuous spectrum. Lastly, for the visuospatial z-scores from games and ACE-III we observe a negative correlation value − 0.0117. The negative and low-magnitude correlation could be due to cases which got high score in ACE-III VS tasks, but compared to this high performance their game-based VS score was low. The paired t-testing is not statistically significant (Mean Difference =0, 95% CI (− 0.313, 0.313)), p-value approximately 1. Thus, the null hypothesis (Eq. 3) cannot be rejected. The 95% limits of agreement for z-scores are shown in Fig. 2d and range from $-$2.78 to 2.78.

From the results of the paired t-test it is clear that there is no statistically significant difference in the z-scores of the ACE-III and game-based scores. Thus, game-based cognitive assessment tools developed by us can be potentially useful. In contrast to previous analytic methods which focus only on correlation; we conducted multiple comparative analyses because we did not want to base conclusions on single analysis. We examine the data for paired difference in the z-scores values and also find out the limits of agreement. Both these methods inform us much more objectively about the scores from two different modalities unlike correlation which provides very limited insights about the scores.

In the next section, we present and discuss results from our final linear regression analysis.

### Linear regression

To understand the association of game-based scores with factors linked to cognitive health, we created eight linear regression models. The scores regressed were Final Navigation Score (FNS), Final Hand-Eye Game Score (FHS), Navigation Game Attention (NGA), Hand-Eye Game Attention (HEGA), Fly Score (FS), Semantic Memory Retrieval Score (SMRS), Visual Short-Term Memory Retrieval Score (VSTMRS) and ACE-III total. The results are presented in Table  4.

**Table 4:** Tabulation of results of Multiple Linear Regression on Final Navigation Score (FNS), Final Hand-Eye Score (FHS), Semantic Memory Retrieval Score (SMRS), and Visual Short-Term Memory Retrieval Score (VSTMRS), Fly Score (FS), Navigation Game Attention (NGA) and Hand-Eye Game Attention (HEGA) using the common variables. Significant values are in bold.

Variable	FNS	FHS	SMRS	VSTMRS	FS	NGA	HEGA	ACE-III
Demographic Factors								
Age	$-$0.72(0.5)	$-$1.00 (0.15)	$-$0.98 (0.22)	$-$0.76 (0.30)	$-$0.07 (0.10)	$-$0.008 (0.33)	$-$**0.006 (0.02)**	$-$0.057 (0.76)
Gender (Male/Female)	3.13 (0.23)	**10.11 (0.007)**	$-$2.94 (0.49)	$-$**10.16 (0.01)**	$-$0.028 (0.90)	0.006 (0.87)	$-$**0.03 (0.009)**	$-$0.606 (0.54)
Lifestyle Factors								
Sleep Duration	$-$1.22 (0.18)	$-$0.48 (0.70)	$-$1.20 (0.42)	$-$0.99 (0.46)	$-$0.131 (0.13)	$-$0.01 (0.32)	$-$0.002 (0.61)	0.013 (0.97)
Sleep Quality	0.31 (0.57)	1.08 (0.17)	1.09 (0.24)	0.86 (0.31)	$-$0.06 (0.24)	0.01 (0.34)	0.00 (0.79)	0.158 (0.467)
Alcohol Consumption	** 5.52 (0.038)**	5.56 (0.14)	0.46 (0.91)	$-$7.2 (0.07)	0.45 (0.07)	0.076 (0.089)	0.02 (0.07)	1.84 (0.073)
Coffee	$-$3.51 (0.066)	2.03 (0.44)	$-$0.24 (0.94)	$-$1.00 (0.72)	$-$**0.59 (0.00)**	$-$**0.06 (0.048)**	0.002 (0.84)	0.46 (0.517)
Smoking	$-$3.95 (0.48)	$-$**17.08 (0.03)**	$-$12.72 (0.167)	$-$1.55 (0.85)	$-$0.47 (0.38)	$-$0.06 (0.50)	$-$**0.14 (<0.0001)**	$-$**4.61 (0.027)**
Social Interaction	$-$1.52 (0.16)	$-$0.85 (0.57)	$-$1.48 (0.41)	$-$1.01 (0.53)	$-$0.089 (0.39)	$-$0.018 (0.31)	$-$0.00 (0.90)	0.006 (0.987)
Game-Based Factors								
Digital Usage	$-$0.005 (0.99)	2.42 (0.40)	$-$1.11 (0.73)	2.37 (0.43)	$-$0.20 (0.30)	$-$0.015 (0.66)	0.007 (0.50)	NA
Gaming Confidence	2.02 (0.17)	$-$1.98 (0.34)	1.32 (0.59)	1.27 (0.57)	0.19 (0.18)	0.047 (0.062)	0.001 (0.86)	NA
VR Experience	$-$0.63 (0.47)	$-$2.07 (0.10)	NA	NA	$-$0.01 (0.89)	$-$0.01 (0.32)	$-$**0.02 (<0.0001)**	NA
Mindfulness Factor								
Observe	$-$0.04 (0.77)	0.188 (0.35)	0.22 (0.34)	0.006 (0.97)	0.00 (0.96)	0.00 (0.49)	** 0.001 (0.03)**	0.024 (0.644)

Adjusted for other factors, age was associated with all the game-based scores negatively but was statistically significant in the case of attention scores obtained from Hand-Eye coordination game (HEGA) (coefficient value = − 0.006, p-value = 0.02). The overall negative sign for the age coefficient across the game scores indicates that the game-based scores formulated in our study could capture the age-effects. Given ageing is linked to cognitive decline^128,129^, our finding is positive as it hints at the possibility of capturing age-based cognitive decline through game-based scores.

Males scored higher in the Final Navigation Score (FNS), Final Hand-Eye Score (FHS) and Navigation Game Attention (NGA), but females scored higher in the Semantic Memory Retrieval Score (SMRS), and Visual Short-Term Memory Retrieval Score (VSTMRS), Fly Score (FS), Hand-Eye Game Attention (HEGA) and ACE-III total score. In the case of FHS, males scored 10 points more than females in the Hand-Eye Coordination game (coefficient value = 10.11, p-value = 0.007) but obtained lower attention scores (HEGA) than females (coefficient value = − 0.03, p-value =0.009), adjusted for other factors. Also, males scored 10.16 points less than females in the Visual short-term memory game (p-value = 0.01). It appears that male participants could perform well in the action related tasks, but in the memory and attention tasks, they scored lower than the female participants. Thus, our game-based scores could also capture the gender differences in the data and are aligned with previous research which reports gender effects on memory^130,131^ and attention^132^.

Sleep duration was not found to be statistically significant with any of the game-based scores, however its coefficient’s sign was negative for all of them. Higher sleep duration was negatively associated with the game-based scores but positively with the ACE-III total. This finding is inconsequential to make any categorical conclusion and more data are required. Sleep quality was found to be positively associated with all the game scores and also ACE-III scores, but was not statistically significant for any of them. Given the data we have, the positive impact of sleep quality on cognitive abilities is hinted at; similar findings are reported in literature^133–136^. However more data are required to categorically establish these results.

The coefficient for alcohol consumption was positive for all the scores and statistically significant for the Navigation game total (FNS) (coefficient: 5.52, p-value = 0.038). As the participants in the study reported once a month alcohol consumption which can be considerate moderate, the coefficient of alcohol consumption illustrate that moderate drinking was associated with better attention and planning and processing speed (the main constituents of the FNS score). Previous cross-sectional and longitudinal research confirm this^137^. The coefficient for alcohol consumption was negative and marginally significant for VSTMRS ($-$7.2, p-value = 0.07), hinting that moderate alcohol consumption may be linked to poor short-term memory. Previous research also confirms that even small amounts of alcohol impairs memory^138,139^. Coffee consumption was found to be associated with lower Visuospatial Scores (− 0.59, p-value   0.00) and Navigation game-based attention (NGA) scores (− 0.06, p-value = 0.048). This association can be random as coffee consumption has been found to be associated with improved cognition^140^

It is confusing to notice that moderate alcohol consumption was associated with improved game performance in the navigation game but not in the memory game. Given that this study was not a randomized controlled trial exploring the impact of alcohol and coffee consumption on cognitive abilities, the findings could be random. Still, we wanted to adjust for these factors to obtain an clear association of cognition-influencing factors with game-based cognitive scores. For both alcohol and coffee consumption, the frequency and amount of consumption are vital to obtain more informed associations. Also, direct evaluation studies, such as randomized controlled trials, pre-post experimental studies, and longitudinal studies, may be more reliable. In the context of our work, we urge readers to interpret our results from an association point of view and appreciate the use of these factors for confounding adjustment.

Smoking coefficient was found to be negative for all the game-based scores and also for the ACE-III total. The coefficient was statistically significant for the FHS ($-$17.08, p-value = 0.034) and HEGA (− 0.14, p-value < 0.0001). These scores belong to the Hand-Eye coordination game which requires sustained attention and quick response. It appears that participants who smoked had lower ability to sustain attention and their hand-eye coordination abilities were compromised. Thus, scores from the Hand-Eye coordination game were responsive to the smoking behaviour which is associated with lower response time and attention^141–145^. Social interaction reported in the sample could not explain any variance in the game-based scores or the ACE-III scores because majority of the participants (78%) were highly socially active and other 18% reported average level social interaction.

To compare the results of the game-scores regression with a standard, we regressed ACE-III on the cognitive health factors. The model was not adjusted for the gaming related variables as these variables are not directly relevant to ACE-III performance. Statistical significance was found for smoking and the coefficient value showed that those who smoked scored 4.61 points lesser than those who did not (p-value = 0.0273). In addition, marginal statistical significance was also found for alcohol consumption and ACE-III (coefficient value: 1.84, p-value = 0.073). Regression on the game-based scores could also capture the associations relating to alcohol consumption and smoking behaviour. After z-score validation, our game-based scores are further validated by the results of the regression analyses.

In particular, scores from the Hand-Eye coordination game like the Final Hand-Eye score (FHS) and attention score (HEGA) have successfully captured the association with age, gender, smoking - factors that are linked to cognitive health, after adjusting with other factors. Thus inspiration from similar games can be taken for designing games for cognitive and functional assessment. Noteworthy is the statistical significance of HEGA score with age (coefficient = − 0.006, p-value =0.02). This is the only score out of all the game and non-game based scores (ACE-III) which reported statistical significance and validates the HEGA score. This can be attributed to the game-play of the hand-eye coordination game. Unlike the Navigation game which involved moving VR controller buttons for movement in the environment, the hand-eye coordination game involved the entire body movement, including the hands and arms. Due to this coordinated movement of the body as a whole in the virtual environment, the game was very similar to performing tasks in the real-world. Thus, the entire game was extremely ecologically congruent with the real-world actions and decision making, therefore the scores obtained from the game were highly correlated with the primary factor associated with cognitive decline (age), after adjusting for other factors.

Thus it appears that games which involve body as a whole are more reliable predictors of cognitive ability because they provide the opportunity to closely engage in real-world actions, movements, coordination, decision-making and perception. This unique ability of such games address the ecological validity limitation inherent in the traditional neuropsychological tests. Therefore, for goal-oriented games to be used as markers of cognitive abilities and early cognitive deterioration, it is essential that realism in virtual environments is equated not only with the game environment and design but also with the nature of interaction with the environment. As observed in our study, games that involve movements of limbs akin to the real-world are more realistic and thus, more reliable for ecologically valid cognitive assessment. Games in themselves may be realistic, but unless they also involve use of brain and body in a realistic and naturalistic manner, they may not be able to aid in cognitive assessment, as observed in our Navigation and Memory games.

To introduce more realism in the VR environments, researchers have started using VR gloves instead of controllers. Such gloves provide haptic feedback and introduce deeper realism while engaging with the VR^146,147^. In future, it will be very interesting to experiment with the games using VR gloves. Currently, VR controllers have limitations because they are far removed from how hands are used in real world and it is difficult for the elderly to learn and remember how to use the controller buttons^148^. Thus, VR controllers cause barrier in naturalistic engagement with the VR-games. In our hand-eye coordination game, VR controller buttons were not used to play the game, the controllers were simply held as hammers in the respective hand. Thus, intuitive hand movements while holding the controller and full body movement during the game helped in tapping into the cognitive abilities in a superior way, as opposed to the Navigation and Memory games. Thus, games that *embody* cognitive decision making are more reliable for assessing real-world cognitive abilities.

Although the study was conducted in the healthy young cohort, the findings are encouraging and motivate similar pilot at larger scale including the older group. Insights from both the young and old cohort would provide deeper clarity on which games to use and develop for cognitive assessment. Such a comparative research will also aid in answering questions linked to scalable solutions for early detection of cognitive deficits. Next, we discuss our findings in the context of previous research.

Evidence linked to the use of goal-based brain games for cognitive performance assessment abound. In a study of 134 participants, game performance correlated with the traditional neuropsychological assessments for fluid reasoning, visuospatial abilities and processing speed^53^. The same study also found that the cognitive processes underlying games and neuropsychological assessments are shared. From these findings it is clear that games engage the cognitive faculties of the player and the scores obtained from them could be used to estimate cognitive functioning. Thereby providing alternative ways to measure cognitive abilities. In another research among young undergraduates and post-graduates, Stroop task scores were found to be correlated with the scores on game platform (Montaj). Based on this finding, authors recommended Montaj for cognitive assessment and remarked that the engaging and enjoyable aspect of the games incites willingness to participate^149^. Although these studies testify the potential of games for cognitive assessment their verification was limited to correlation-based analyses whereas we have provided a detailed comparative approach based on z-scores, paired t-testing, limit of agreement, and regression analyses.

Apart from the quantitative analyses, we have also leveraged on the granularity of the data obtained from the games and used it to understand unique cognitive traits of the players. Games are effective in capturing the unique decision making or functional abilities of the players which is vital for differentiating true cognitive decline from the perceived cognitive decline. Therefore, games can provide more ecologically valid assessments of cognitive and functional abilities. Even in the recruitment decisions, VR-based intelligence assessments^150^ which can simulate real-world situations^151^ have been found helpful due to their higher level of standardization^152^. Still, authors based their analysis purely on correlation analysis and on obtaining weaker correlation concluded that VR-tools can be use for pre-screening^153^.

In our work we deliberately refrained from basing validation exclusively on single analysis, like the correlative analyses, because it provides a narrow perspective on the games’ efficacy and downplays other advantages of game-based assessments like granular data, immersive environment, contextual evaluation, and real-world cognitive load simulation. All of these advantages are highly relevant in minimizing the gap between obtained game scores and actual behaviour in real world. Furthermore, studies that establish correlation as the sole factor to validate cognitive games fail to discuss the real-world and clinical implication of the correlation values. Still, understanding correlative analyses as the starting point, we undertook it in our work. However, we further explored the deeper interaction between the game-based scores and traditional scores using statistical analyses on the respective z-scores. We also attempted to explore the implication of the game scores in real world, by regressing them on factors linked to cognitive decline. Thus, our approach and attitude to the game-scores validation is far more nuanced, diverse and informative.

A possible criticism of our work could be our young sample, but we believe it is a valid starting point as some aspects of age-related cognitive decline begin early in the 20s or 30s^11^. Moreover, given the traditional post-symptomatic approach to cognitive decline diagnosis, our study emphasizes taking early accountability and is aligned with preventive and life-course approach to cognitive decline. Still, research among older participants, both healthy and non-healthy, would lead to more clarity on the obtained results.

Another limitation of our sample is that it did not have MCI people. But, it is important to point out that previous studies on VR game-based cognitive assessment and rehabilitation have also based their findings on young sample. For example, 60 participants, mean age 20 years were tested using a VR game which aimed to train them in executive functioning skills^97^. Similarly, navigation games in VR were shown to differentiate between good and poor navigators based on a sample of 32 participants with mean age of 23 years^69^. Feasibility of VR based games for executive function enhancement was tested in 16 participants with mean age 21 years^98^. 15 university students mean age 27 years were tested in a virtual hand controlled task and their performance was compared using fMRI^67^. Similar other studies have based their findings on the efficacy of VR-based tools by testing in the young participants^62,65,66,100,119,154^.

While, the importance of using older participants both health and MCI is very important to establish the utility of the VR-based cognitive assessing games^61,68,99,101–103^, it is important to acknowledge the difficulty associated with identifying and recruiting such participants. Normally, a research institution has to partner with either hospitals, clinics, medical colleges, or NGOs to enter into a formal agreement for research. This can be a long process depending upon the protocols of the organization providing the older or MCI participants. Furthermore, in India, unlike in foreign universities, medical colleges are separate and technical colleges are separate. Therefore, practical feasibility challenges exist in recruiting clinical population. Given these realities, in our initial experiment on VR and Mobile-based 3D games we chose a younger group for testing. This also allowed us to identify the software issues in the games. Prior evidence on age-related cognitive decline also encouraged us to undertake this experiment.

Nevertheless, mindful of the limitation of our study, our entire research approach was based on identifying whether or not the game-based cognitive scores were associated with the factors linked with cognitive impairment. Instead of strictly testing the games for their screening or diagnostic ability, we wanted to assess whether they could capture the gradient associated with cognitive decline. We believe that if we have to approach the problem of early detection of cognitive impairment, we have to transcend the rigid approach of healthy and diseased and instead look at cognitive health and impairment as a gradient. Through such an approach, it would be easier detect cognitive red flags earlier by comparing the player with the age-gender matched population statistics.

## Conclusion

In this work, we presented and discussed the results of the pilot study on using 2 VR and 1 Tablet-based games for cognitive assessment among 18–28 year old participants. The z-score analysis on the game and Addenbrooke’s Cognitive Examination (ACE-III) scores did not show any statistically significant difference indicating comparability of the two assessment modalities, however lower correlation and higher 95% limits of agreement were observed. Regression analysis found superior statistically significant association between cognitive health factors like age, gender and smoking and Hand-Eye Coordination game scores, than for ACE-III. Thus, Hand-Eye coordination game may have potential to capture the cognitive abilities and deficits. However, it needs to be tested in the older cohort to substantiate this finding.

Specific observations from the participants’ game scores revealed their unique cognitive traits, thereby hinting at the possibility of ecologically valid insights from game scores. Overall, game-based z-scores were found aligned with the ACE-III z-scores, which was used as a traditional neuropsychological assessment counterpart. More human-based clinical research is required to develop age-based norms for the game-based cognitive assessments, before integrating them into mainstream neuropsychological assessment. Even so, game-based cognitive assessments have enough evidence to promote their usage for monitoring and measuring specific cognitive and functional abilities.

## Supplementary Information

Supplementary Information.

## Declarations

### Data availability

Currently, data are being used for different research purposes, so they cannot be shared openly. Still, inquiries can be directed to the corresponding author.
