# Supplementary material for: Validation and comparison of virtual reality and 3D mobile games for cognitive assessment against ACE-III in 82 young participants
Source: Sci Rep. 2024 Oct 13;14:23918. doi: 10.1038/s41598-024-75065-1 (PMC11471807; doi:10.1038/s41598-024-75065-1)
Supplement: Supplementary file 1 — Supplementary Information. [file 41598_2024_75065_MOESM1_ESM.pdf]

Supplementary Materials

Navigation Game

In this course-obstacle game (Figure 1) the participant has to wear the VR headset and use the controllers to travel (walk/fly) through an animated virtual world course (land and sky), collect coins (rewards), identify turning points and avoid obstacles to reach the final treasure. In the fly-course, the participant uses controllers to fly and collect the coins in the hoops suspended in the air (Figure 2).

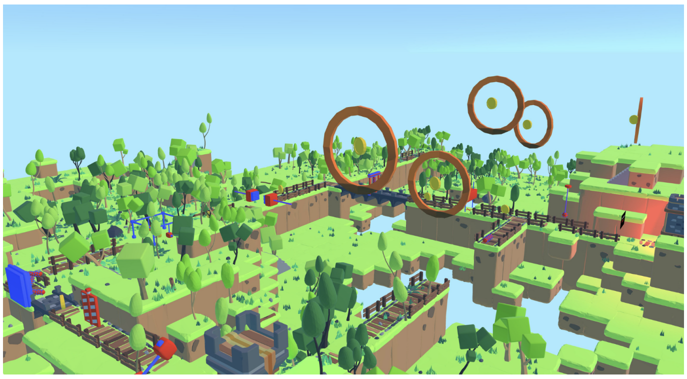

Fig. 1 Navigation Game Sky-view. The snapshot is taken from the Navigation game originally developed by us. Software used : Unity (Version 2022.3).

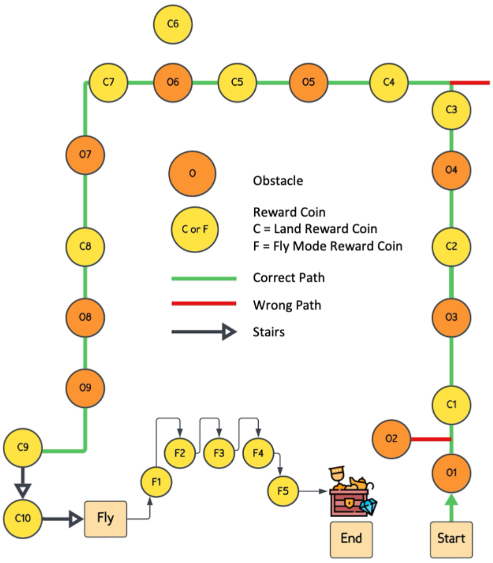

Fig. 2 An abstraction of the VR-based Navigation game showing the Obstacles, Rewards (on Land and in Fly section), Correct and Incorrect paths and the Fly section. Figure made in Lucidchart.

Cognitive scores for the Navigation game were computed based on the constructs of Processing Speed, Attention and Planning, Navigation Ability and Visuo-Spatial ability developed by us. These constructs were conceptualized and formulated based on the understanding of the cognitive skills required during the game. Similar approach is reported in previous work on VR-based cognitive assessment [1]. The scores are described below:

Processing Speed ***(PS_n)***

The number of obstacles successfully crossed (no collision) per unit time is formulated as the PS_n. In total there are 8 obstacles which form the part of the land-course game whereas one is an off-track obstacle and not counted for computing PS_n (Figure 2). Equation 1 shows the formula used to compute this score; the subscript n denotes that the score corresponds to the Navigation game.

$$PS_n = \left(\frac{\text{Total Obstacles successfully crossed}}{\text{Game Finish Time}} \right) \tag{1}$$

Attention and Planning ***(AP_n)***

For this score, we used the weighted average of the time taken to cross each obstacle. Given there were a total of 8 obstacles $(O_1, O_2, O_3, O_4...., O_8)$, with complexity $(C_1, C_2, C_3, ..., C_8)$ and out of these m were crossed successfully where $m <= 8$, and p were not ($m + p = 8$). Suppose, the time taken to overcome each of these m obstacles is $(t_1, t_2, t_3, ...t_m)$ then the Attention and Planning score was computed as shown in Equation 2.

$$AP_n = \left(\frac{\Sigma_{i=1}^{m} C_i * t_i}{\Sigma_{i=1}^{m} t_i} + \frac{1}{\Sigma_{i=1}^{p} C_i} \right)^{-1} \tag{2}$$

The component $\frac{1}{\Sigma_{i=1}^{p} C_i}$ provides penalty to the AP_n score and is added only when $p > 0$. Accordingly, a higher penalty is given when the complexity of the unsuccessfully avoided obstacle is less and vice-versa. In the Navigation game, the complexity for the 8 obstacles $(O_1, O_2, O_3, O_4, ..., O_8)$ was decided to be (1, 2, 1, 2, 1, 3, 2, 2) respectively. These obstacle complexities were decided based on the relative difficulty to overcome these obstacles.

Navigation Ability ***(NA_n)***

To compute this score, we used the time spent by the participant at each turn in the game path. In the VR environment, there are total 3 turns (green blobs in Figure 2) and 2 wrong paths (red lines in Figure 2). Because the wrong path does not lead to the treasure, the player must avoid them, or quickly act to come back on the right-course in case they enter the wrong path. The navigation score was computed as the inverse of the total time spent in wrong paths (red lines) and time taken to make the turns (green blob) (Equation 3). Such a score is important because identifying paths and turns is important life skill [2] and its deficit is linked to early detection of AD and amnestic mild neuro-cognitive disorder [3].

$$NA_n : \left(\frac{\text{wrongPathTime} + \text{turningPointTime}}{60}\right)^{-1} \tag{3}$$

Visuo-spatial Score ***(*VS_n*)***

This score was computed based on the performance in the fly segment of the Navigation game (Figure 2) because this segment provided greatest opportunity to test the VS ability. In the fly segment, the player has to identify the direction in which hoops are hanging and direct the motion of the controllers to collect the coins in those hoops. At the end of fly segment, the player reaches the treasure point (Figure 2). Equation 4 shows the formula used to compute VS_n.

$$VS_n = \left(\frac{\text{Fly Coins Collected}}{\text{Total Fly time}}\right) \tag{4}$$

Higher values for PS_n, AP_n, NA_n and VS_n indicate better scores. Based on these scores, two final scores for the Navigation game were computed : Land Score (Equation 5) and Fly Score (Equation 6).

$$\text{Land Score} = PS_n * AP_n * NA_n * \text{Total land coins collected} \tag{5}$$

$$\text{Fly Score} = VS_n * \text{Total fly coins collected} \tag{6}$$

Hand-Eye Coordination Game

In the second VR game, the player is expected to hit blue cube by the blue hammer (VR controller) and the red cubes by the red hammer (Figure 3). The blue hammer is in the left hand of the player, and the red on is on the right hand. A correct hit is registered when the red/blue cube is correctly hit by the red/blue hammer respectively, in the direction specified on the incoming cube. Any other hit is incorrect. With every correct hit, the speed of the incoming cubes increases. The game ends when the player hits 50 cubes or when 150 seconds are over, whichever is earlier.

Based on the game performance, scores on Processing Speed, Attention Right-Hand, Attention Left-Hand, and Motor Abilities are computed. Mathematical formulation of these scores is described next. The subscript h serves as an identifier to the Hand-Eye Coordination game.

Processing Speed ***(*PS_h*)***

Computed as the total number of correctly hit cubes per unit time (Equation 7). PS_h quantifies the quickness of the player in response to the incoming cubes (stimuli).

$$PS_h = \left(\frac{\text{Total correctly hit cubes}}{\text{Game duration (in seconds)}}\right) \tag{7}$$

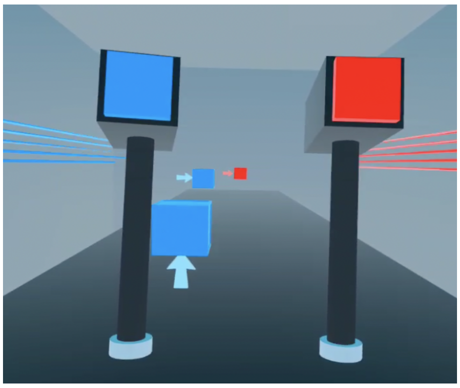

Fig. 3 Hand-Eye game first-person view. The snapshot is taken from the Hand-Eye coordination game originally developed by us. Software used : Unity (Version 2022.3).

Attention scores

Given there are two types of cubes in the right (red) and left (blue) sides, attention score is computed for both the left and the right hands. These are called the Attention Right Hand (ARH_h, Equation 8) Score and Attention Left Hand (ALH_h, Equation 9) score.

$$ARH_h = \left(\frac{\text{Correctly hit red cubes}}{\text{Total incoming red cubes}}\right) \tag{8}$$

$$ALH_h = \left(\frac{\text{Correctly hit blue cubes}}{\text{Total incoming blue cubes}}\right) \tag{9}$$

Motor Abilities

The incoming cubes come in four directions : Right, Left, Up and Centre. To hit the cubes in these directions, sometimes the player has to move sideways (right/left) or raise hand over the head. Thus, movement of lower limbs and upper limbs is required during the game. We capture these movements through the Motor Abilities score for all four directions (Equation 10, 11, 12, 13). The score is based on the attempted hit to the cube, which signals that limb movement was made to reach out to the cube. Thus, a cube my be hit correct or incorrectly, but in both the cases the attempt is counted as valid and used to compute these scores. Such scores can potentially inform about cognitive deterioration as they are linked to early cognitive decline [4].

$$\text{Motor Right} \;=\left(\frac{\text{No. of right-side cubes attempted}}{\text{Total incoming right-side cubes}}\right) \tag{10}$$

$$\text{Motor Left} \;=\left(\frac{\text{No. of left-side cubes attempted}}{\text{Total incoming left-side cubes}}\right) \tag{11}$$

$$\text{Motor Up} \;=\left(\frac{\text{No. of upward cubes attempted}}{\text{Total incoming upward cubes}}\right) \tag{12}$$

$$\text{Motor Centre} = \left(\frac{\text{No. of centre cubes attempted}}{\text{Total incoming centre cubes}}\right) \tag{13}$$

For the Hand-Eye coordination game, data on total number of missed/wrongly hit cubes was also stored.

Total Hand-Eye Score

Finally, we computed the total Hand-Eye score. We conceptualized Total Attention (TA) and Total Motor (TM) scores for this, which are explained below.

Let a (ALH_h) and b (ARH_h) be the attention scores as defined in the equation 9 and 8 respectively. The Total Attention Score for the hand-eye coordination game is defined as:

$$\text{Total Attention (TA)} = \left(\frac{a+b}{2}\right) \tag{14}$$

Let r, l, c, u be the motor score proportions as defined in the equation 10, 11, 13, and 12 respectively. Then, we define Total Motor Abilities score for the hand-eye coordination game as:

$$\text{Total Motor (TM)} = \left(\frac{r+l+c+u}{4}\right) \tag{15}$$

Thus, the total Hand-Eye Score is given by Equation 16 when there are non-zero wrong hits otherwise equation 17 is used .

$$\text{Hand-Eye Score} = PS_h * TA * TM * \frac{1}{\text{Total Wrong Hits}} \tag{16}$$

$$\text{Hand-Eye Score} = PS_h * TA * TM \tag{17}$$

Memory Games

The Memory game is laptop/tablet based and consists of 4 levels (Figure 4). The first two levels require the subject to spot and tap on the irrelevant objects in office (Figure 4(a)) and kitchen (Figure 4(b)) environment. The next two levels require the subject to spot the objects that change color (Figure 4(c)) or disappear from the kitchen table (Figure 4(d)) after a timer of 10 seconds. For each of these four levels, two individual scores were designed : Recall (correct answers retrieved out of total correct answers) and Precision (proportion of successful retrievals out of total number of attempts).

A final Memory score was created using the harmonic mean of these two scores (Table 1). Additionally, a Memory Speed score was computed based on the time taken to finish each level (Table 1). As the first two levels test semantic knowledge, corresponding scores are called the Semantic Memory Retrieval (SMR) score, and Semantic Memory Retrieval Speed (SMRS) score respectively. In the third and fourth level, short-term memory is tested, hence the corresponding scores are called

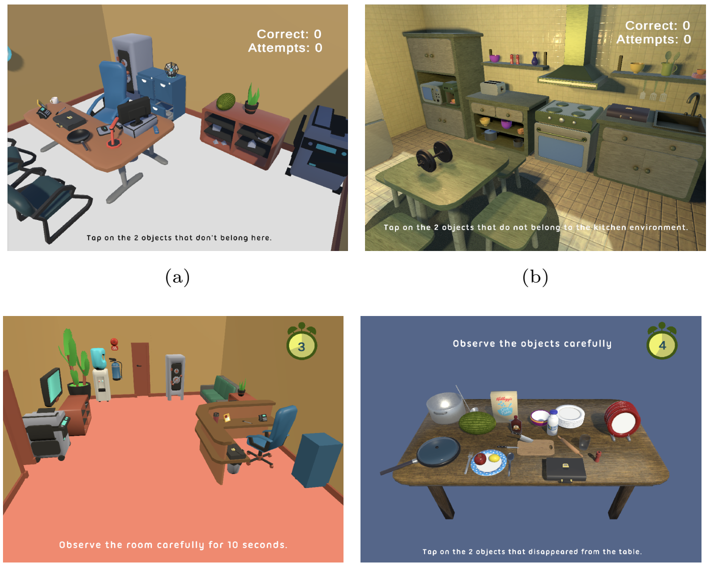

(a) (b) (c) (d)

Fig. 4 Semantic Memory game environments. (a) : Office Environment. (b): Kitchen Environment. (c): Color Change. (d): Object Disappearance. The snaphots are taken from the memory games originally developed by us. Software used : Unity (Version 2022.3).

Table 1 Memory Score Details

Attempts	Successful Retrieval	Recall	Precision	Memory Score	Game finish time	Memory Score Speed
2	2	1	1	1	t_1	$1/t_1$
3	2	1	0.6666666667	0.8	t_2	$0.8/t_2$
4	2	1	0.5	0.6666666667	t_3	$0.67/t_3$
5	2	1	0.4	0.5714285714	t_4	$0.57/t_1$
5	1	0.5	0.2	0.2857142857	t_5	$0.286/t_1$
5	0	0	0	0	t_6	NA

the Visual-Short Term Memory Retrieval (VSTMR) score and Visual Short-Term Memory Retrieval Speed (VSTMRS) score.

Next, we explain in detail the score formulation approach for the memory games. Each of the 4 levels for the memory game consisted of only 2 correct answers. Participants had 5 attempts to identify these 2 answers. Accordingly, the best case would be 2/2, i.e. 2 attempts and 2 successes in those attempts and the worst-case scenario would be 0 out of 5 attempts. Based on this, we identified all combinations of attempts and correct answers (Table 1). For each of these combinations, Recall and Precision was computed, which was used to compute the Memory Score (SMR and VSTMR). Using the time taken to finish the levels, the Memory speed score was computed (SMRS and VSTMRS).

For each participant the total Semantic Memory score was computed as the sum of the product of SMR and SMRS from level 1 and 2. The total Visual Short-Term Memory score was computed as the sum of the product of VSTMR and VSTMRS

from level 3 and 4. These total Semantic Memory and Visual Short-Term Memory score were added to indicate the total game-based Memory score. This score served as comparative counterpart to the ACE-III memory score.

References

[1] Tarnanas, I.A., Schlee, W., Tsolaki, M., Müri, R.M., Mosimann, U.P., Nef, T.: Ecological validity of virtual reality daily living activities screening for early dementia: Longitudinal study. JMIR Serious Games **1** (2013)

[2] Hegarty, M., Montello, D.R., Richardson, A.E., Ishikawa, T., Lovelace, K.L.: Spatial abilities at different scales: Individual differences in aptitude-test performance and spatial-layout learning. Intelligence **34**, 151–176 (2006)

[3] Hort, J., Laczo, J., Vyhnalek, M., et.al.: Spatial navigation deficit in amnestic mild cognitive impairment. Proceedings of the National Academy of Sciences **104**, 4042–4047 (2007)

[4] Willik, K.D., Licher, S., Vinke, E.J., Knol, M.J., Darweesh, S.K.L., Geest, J.N., Schagen, S.B., Ikram, M.K., Luik, A.I., Ikram, M.A.: Trajectories of cognitive and motor function between ages 45 and 90 years: A population-based study. The Journals of Gerontology Series A: Biological Sciences and Medical Sciences **76**, 297–306 (2020)
